# Validation of the cultural adaptation of the Kujala score in Arabic

**DOI:** 10.1186/s13018-021-02489-0

**Published:** 2021-05-19

**Authors:** Bassem I. Haddad, Mohammad Hamdan, Ula Isleem, Munther Ghassan Al-Saber, Fadi A. Al-Hadidi, Saif Aldeen AlRyalat, Fatima Alnaimat

**Affiliations:** 1 Department of Special Surgery, School of Medicine, University of Jordan, Queen Rania Street, Amman, 11942 Jordan; 2 School of Medicine, University of Jordan, Queen Rania Street, Amman, 11942 Jordan; 3 Department of Internal Medicine, School of Medicine, University of Jordan, Queen Rania Street, Amman, 11942 Jordan

**Keywords:** Cultural adaptation, Kujala score, Patellofemoral pain, Arabic translation

## Abstract

**Background:**

Patellofemoral pain is a common condition. The Kujala score is a well-established scoring system to assess anterior knee pain and has been translated into many languages including Arabic. The purpose of this cross-sectional study is to culturally adapt the Arabic version of the Kujala score and determine its validity.

**Methods:**

The Kujala score is composed of 13 multiple-choice questions. We modified two questions in the score; running and squatting, and were replaced with questions related to walking on different terrain and prostration, each with the same number of answer choices as the original questions so as not to affect the final score. These modifications were written in Arabic by the same group who translated and validated the original score into Arabic. The original and modified Kujala scores was printed and given to patients complaining of patellofemoral pain to be filled during their visit to the orthopedic outpatient clinics.

Final scores for the original and modified questionnaires were calculated. Data was analyzed using SPSS statistics version 21.0 measuring Cronbach’s alpha, intraclass correlation coefficient, and Pearson correlation.

**Results:**

Ninety-four patients were included in the study, 28 (29.8%) men and 66 (70.2%) women. The mean age for the included patients was 43.67 (± 14.46) years. The mean score for the modified initial questionnaire was 63.91 (± 16.32), and the mean score for the modified re-test questionnaire was 66.52 (± 17.50). There was a statistically significant difference between the mean scores (p = 0.041), with a mean difference of 1.97 (95% CI 0.08 to 3.856).

We found a significant strong correlation between the score before and after changing the questions with a p value of < 0.001.

**Conclusions:**

The culturally modified Arabic Kujala questionnaire is shown to be a valid, well-designed tool and an appropriate method of measuring patellofemoral pain.

## Background

Patellofemoral pain syndrome (PFPS) is a common problem affecting the knee joint, with an incidence of 10–20% in the general population [1]. PFPS is usually observed in adolescents and adults under the age of 60 years who engage in activities that would exert additional load to the patella-femoral joint, which include and are not limited to using the stairs (especially descending motion), running, squatting, and climbing. Women are affected twice as often than men, and patients usually deny any traumatic events before the onset of symptoms [2].

This disease is attributed to a combination of extrinsic and intrinsic factors. Extrinsic factors include the type of exercise being practiced, its intensity and frequency, and the equipment being used [3]. On the other hand, intrinsic factors include age, gender, the stability of joints, and muscle strength [4]. Suggested modifiable risk factors, including patellar mal-alignment, joint laxity, quadriceps weakness, hamstring and iliotibial band tightness, iliopsoas and gastroc-soleus muscle dysfunction, are of paramount importance in developing PFPS [5].

There is no definitive gold standard method to diagnose PFPS. The diagnosis is mainly clinical. Pain elicited on a number of tests including the patellofemoral compression test and resisted knee extension, along with a properly taken history aid in diagnosis [1]. Assessment of the patient’s gait, lower limb alignment, Q-angle, femoral anteversion, and knee range of motion should not be neglected as well to rule out any other pathology [6].

Treatment usually revolves around conservative management in the form of physical therapy (which concentrates on increasing the strength and flexibility of the quadriceps femoris muscle (mainly the vastus medialis obliquus muscle)) and analgesia [6].

The aim of this study is to culturally adapt the Arabic version of the Kujala score to better accommodate the local population's culture and activities. We hypothesized that this culturally adapted score is valid and reliable in assessing patients with PFPS.

## Methods

The appropriate institutional review board (IRB) approved the proposal for this study. The Code of Ethics of the World Medical Association (Declaration of Helsinki) was followed while conducting the study. All patients participating in this study signed a written informed consent.

The Kujala score is composed of 13 multiple choice questions: the presence of a limp, the need for support, the ability to walk, the ability to climb stairs, the ability to squat, the ability to run, the ability to jump, prolonged sitting with knees in the flexed position, the presence of knee pain, the presence of knee swelling, the presence of abnormal painful patellar movement, atrophy of the thigh muscles, and deficiency of knee flexion. We modified two questions in the score; the abilities to run and jump, and were replaced with questions related to walking on different terrain and prostration, each with the same number of answer choices as the original questions so as not to affect the final score. These modifications were written in Arabic by the same group who translated and validated the original score into Arabic [7].

Two questions were substituted with questions that better reflect the activities done by the average Jordanian citizen. The Jordanian population is not well known for certain activities including running and jumping (which are both essential components of the score). Therefore, implementing these points in the final score may give a false impression about the disease severity. Question number six “Running” was changed to “Walking on different types of terrain” due to various and uneven ground present in Jordan due to its mountainous nature. Question number 7 “Jumping” was changed to “Prostration” as the majority of Jordanians are Muslims who practice the act of “Prostration” several times per day as part of their daily prayers.

The original Kujala score along with the two modified questions (totaling 15 questions) was printed and given to patients complaining of patellofemoral pain to be filled during their visit to the orthopedic outpatient clinics. A member of the research team was present throughout the whole time questionnaires were filled to help patients out or in case any clarification was needed. The number of patients who filled the questionnaire was 127. These patients were contacted by phone by research team members at least 2 weeks later and were asked the same questions. Two total scores were calculated for each patient; the original score (using the 13 original questions) and the modified score (using the score of the two modified questions instead of the original two).

Included patients were diagnosed to have PFPS by history and physical examination (using Clark’s sign and Waldron’s test [1, 8, 9]) performed by either one of the two senior authors (BIH and MH). Patients denied history of knee trauma or surgery and lacked pathological X-ray findings including osteoarthritis, fractures, tumors, osteonecrosis, or Osgood-Schlatter disease. Patients who were lost to follow-up upon re-contacting were excluded.

The final number of included patients was 94. Most of the patients (70.1%) were successfully contacted after at least 2 weeks, while the rest (29.9%) were contacted later. Five patients had started physical therapy sessions at the time of phone contact to re-fill the questionnaire.

### Statistical analysis

Data were entered into and analyzed by SPSS statistics version 21.0 (Chicago, USA). Descriptive statistics were performed on the included patients, and the results were reported using frequency (percentages) and mean (± standard deviation).

Cronbach’s alpha was used for reliability analysis for scale internal consistency. Intraclass correlation coefficient (ICC) was used for reliability analysis for test-re-test analysis. Mean scores for the initial questionnaire and the re-test questionnaire were reported using mean (± standard deviation) and analyzed using paired sample t test and ICC. These were reported using the average coefficient and its 95% confidence interval (CI). The correlation between the score before and after changing questions 6 and 7 was studied using Pearson correlation. A p value of < 0.05 was considered significant.

## Results

The total number of patients included in this study was 94, 70.2% (66 patients) were women and 29.8% (28 patients) were men. The mean age for the total number of patients was 43.67 (± 14.46) years, 45.5 (± 14.64) years for women, and 39.36 (± 13.29) years for men, with no significant age difference between both sexes (p = 0.543). The mean time period between the initial and re-test questionnaires was 12.24 (± 13.38) weeks.

All 94 patients responded to all items of the Kujala patellofemoral score at the first encounter and completed the re-test questionnaire; hence, all were included in the evaluation. The mean total score for the initial questionnaire was 63.91 (± 16.32), and the mean total score for the re-test questionnaire was 66.52 (± 17.50). The difference between the mean scores was statistically significant (p = 0.041), with a mean difference of 1.97 (95% CI 0.08 to 3.856).

A statistically significant correlation was found in the score before and after the proposed modification (p < 0.001) (Table 1).

**Table 1:** Comparison between total score before and after changing questions 6 and 7

		Mean	Standard deviation	Correlation coefficient	p value
Initial questionnaire	Before changing questions	64.77	16.66	0.915	< 0.001
	After changing questions	62.65	17.32		
Re-test questionnaire	Before changing questions	66.73	17.73	0.955	< 0.001
	After changing questions	64.49	18.14		

Scale internal consistency for the modified scale measured via Cronbach’s alpha was 0.806, the score for each of the questions is shown in Table 2. The average ICC was 0.806 (CI 0.742–0.859) for the total score and 0.181 (CI 0.129–0.250) for single measures.

**Table 2:** Total score statistics for individual questions and the contribution of each question to the score

Question number	Score mean if the question was deleted	Score variance if the question was deleted	Corrected question-total correlation	Cronbach’s alpha if the question was deleted
1	62.3936	285.811	.435	.797
2	62.9362	293.931	.280	.804
3	63.6170	276.647	.473	.793
4	60.7553	258.896	.521	.786
5	63.8617	259.282	.720	.775
6	60.2128	236.427	.620	.774
7	60.8404	215.662	.545	.793
8	60.7340	248.972	.486	.789
9	60.1702	263.369	.422	.794
10	57.9468	270.029	.405	.795
11	59.7766	264.950	.359	.801
12	62.1277	297.467	.224	.807
13	62.8830	276.083	.528	.791

Most of the patients (94.7%) were contacted prior to the initiation of physical therapy sessions. After comparing the initial test and re-test total scores, 43 patients (45.7%) showed an improvement (the re-test score was higher than the initial test score), 42 patients (44.7%) showed a mild worsening (the re-test score was lower than the initial test score); and nine patients (9.6%) remained unchanged (equal total scores of the initial and re-test questionnaires).

## Discussion

Our results showed that our cultural adaptation of the original Kujala score is a valid and appropriate tool in assessing patellofemoral pain.

The idea behind this modification was the need for a comprehensive tool to objectively assess patients with PFPS with regards to the severity of various symptoms and the response to treatment. The Kujala patellofemoral score is easy to comprehend [10] and time-efficient [11]. The score is also able to encompass the activities of daily living that are affected by PFPS [12]. Although the Kujala score is thorough and comprehensive, yet some of its aspects may not reflect the status of patients from different cultural backgrounds. In this study, we modified two questions from the Arabic version of the Kujala score to better relate to the region's cultural behaviors.

The original Kujala score was previously translated into multiple different languages to help communicate with different patients. It was translated to Arabic, Spanish, German, Persian, Chinese, and Thai along with other languages [7, 13–17]. Some of these translations were culturally adapted to different practices and activities of daily living that vary among different cultures. We modified two questions of the Arabic Kujala score to better adapt to our culture, and we proposed that these two modified questions would help us better understand the effects of this condition in the Jordanian population.

We thought of the need to modify two questions in the original score, namely questions 6 and 7 which asked about difficulty and pain during running and jumping, respectively, because running and jumping are not a common part of the daily activities of the average Jordanian population. Hence, answering these questions in their original form might not accurately reflect the true symptoms and disabilities of the patients. Two common practices of daily living of our population were considered instead. The first one was walking on different types of terrain considering that Jordan has various terrains and a mountainous nature. Question number 6 was replaced by a question about pain during walking on different terrains (even and uneven). The second was prostration. This is a compulsory practice of the five daily prayers in Islam. Given the fact that 92% of Jordanians are Muslims according to the official site of the Jordanian e-government “(https://portal.jordan.gov.jo/wps/portal/Home/AboutJordan)”, we replaced question number seven by a question about pain during prostration. Both of the modified questions had the same number of answer choices as the original ones.

Our results showed that these changes were useful in monitoring the changes in the patients’ conditions. Cronbach’s alpha was measured after removing each item and varied between 0.77 and 0.8, showing that all the items in the questionnaire were suitable and necessary for its implementation. This observation corresponded to the results of similar studies which translated the Kujala questionnaire to other languages [13–15]. We also found a strong correlation between the results of the original and modified scores. Consequently, our results showed that the translated and culturally adapted questionnaire is a well-designed tool and an appropriate method of measuring patellofemoral pain.

This culturally adapted Arabic Kujala score could be of use in multiple populations including all Arabic-speaking countries in the Middle East and North Africa. If translated into other languages, it could also be used in all Muslim populations, around 1.8 billion people worldwide “(https://www.pewresearch.org/fact-tank/2017/08/09/muslims-and-islam-key-findings-in-the-u-s-and-around-the-world/)”. The now available Arabic version of the Kujala score should facilitate high quality clinical and scientific work in the field of patellofemoral disorders in these populations.

One limitation of this study is the lack of a pre-existing Arabic questionnaire to evaluate patellofemoral pain, limiting studies for comparison to ours. Another limitation is the relatively small number of patients and longer follow-up times in these patients compared to other studies of a similar nature. Finally, no subgroup analysis was performed to compare between patients’ ages despite the wide age range.

## Conclusion

The culturally adapted Arabic version of the Kujala score seems a reliable and valid tool to assess and monitor the clinical progression of patients presenting with patellofemoral pain syndrome.

## Data Availability

All data generated or analyzed during this study are included in this published article [and its supplementary information files].

## Abbreviations

PFPS: Patellofemoral pain syndrome
ICC: Intraclass correlation coefficient
CI: Confidence interval
